# Illumina Amplicon Sequencing of 16S rRNA Tag Reveals Bacterial Community Development in the Rhizosphere of Apple Nurseries at a Replant Disease Site and a New Planting Site

**DOI:** 10.1371/journal.pone.0111744

**Published:** 2014-10-31

**Authors:** Jian Sun, Qiang Zhang, Jia Zhou, Qinping Wei

**Affiliations:** Institute of Forestry and Pomology, Beijing Academy of Agriculture and Forestry Sciences, Beijing, China; Graz University of Technology (TU Graz), Austria

## Abstract

We used a next-generation, Illumina-based sequencing approach to characterize the bacterial community development of apple rhizosphere soil in a replant site (*RePlant*) and a new planting site (*NewPlant*) in Beijing. Dwarfing apple nurseries of ‘Fuji’/SH6/Pingyitiancha trees were planted in the spring of 2013. Before planting, soil from the apple rhizosphere of the replant site (*ReSoil*) and from the new planting site (*NewSoil*) was sampled for analysis on the Illumina MiSeq platform. In late September, the rhizosphere soil from both sites was resampled (*RePlant* and *NewPlant*). More than 16,000 valid reads were obtained for each replicate, and the community was composed of five dominant groups (Proteobacteria, Acidobacteria, Bacteroidetes, Gemmatimonadetes and Actinobacteria). The bacterial diversity decreased after apple planting. Principal component analyses revealed that the rhizosphere samples were significantly different among treatments. Apple nursery planting showed a large impact on the soil bacterial community, and the community development was significantly different between the replanted and newly planted soils. Verrucomicrobia were less abundant in *RePlant* soil, while *Pseudomonas* and *Lysobacter* were increased in *RePlant* compared with *ReSoil* and *NewPlant*. Both *RePlant* and *ReSoil* showed relatively higher invertase and cellulase activities than *NewPlant* and *NewSoil*, but only *NewPlant* soil showed higher urease activity, and this soil also had the higher plant growth. Our experimental results suggest that planting apple nurseries has a significant impact on soil bacterial community development at both replant and new planting sites, and planting on new site resulted in significantly higher soil urease activity and a different bacterial community composition.

## Introduction

Apple trees are among the most important fruit trees in the world. China has the world’s highest apple tree acreage (2.060 million hectares, 42.54% of the world’s supply; FAOSTAT, 2012) and production (37.00 million tons, 48.44% of the world’s supply; FAOSTAT, 2012). However, apple trees in most of China’s dominant production areas experience a full fruit period and senescence phase; currently, approximately 70% apple orchards in China are over 20 years old. The need for apple orchard renewal is more than 140 thousand hectares per year. Complicating this renewal is apple replant disease (ARD), which, because of a lack of land resources, is becoming a serious problem in fruit tree nurseries and old orchards.

The term ARD refers to the poor growth of young apple trees, which occurs after replanting on a site that was previously planted with apple. The phenomenon is common to all major apple growing regions of the world, including Asia, Europe, North America, Africa etc. Compared with new planting sites, directly replanting nurseries on old sites can result in decreased tree growth and a significantly lower seedling survival rate [1]. Trees may take several years to recover from the initial growth depression and eventually reach the size and annual yields of unaffected trees, and the cumulative yields and profitability in ARD-affected orchards are usually much lower than in unaffected orchards [2]. In China, the traditional method to avoid ARD is allowing a fallow period of over three years, while using plants from the grass family such as wheat to ‘clean’ the soil [3]. The efficacy of growing wheat for reducing apple root infection by species of *Rhizoctonia* and *Pythium* was confirmed by greenhouse and field trials [4], [5]. However, the lost productivity is unaffordable for famers because land is limited and expensive.

Typically, in ARD-affected orchards, the root systems of apple seedlings are small, with discolored feeder roots and few functional root hairs [6]. In a previous field trial, leaf analysis for macro- and micronutrients showed most elements in ARD-affected and ARD-unaffected orchards exhibited no significant difference; thus, the observed growth responses were not associated with any nutritional effect [7]. Replant disease of fruit trees has been studied for many decades and although reported to be attributed to certain abiotic elements including phytotoxins [8], nutrient imbalance, low or high pH, and lack or excess of moisture [9], the preponderance of evidence indicates that the disease in large part is due to biotic factors [10]–[12]. Soil sterilization and fumigation with methyl bromide were effective in treating ARD [13], [14], thus demonstrating the role of biotic components of soil as prominent determinants of ARD in orchards.

Soil microorganisms are recognized as the key factor inducing ARD, but it is a challenging task to fully characterize soil microbial communities. The information from culture-dependent methods is limited because only a small fraction of soil microorganisms is culturable [15]. Recently, culture-independent methods have been developed for investigating microbial communities, including molecular analyses of nucleic acids extracted from soil, including denaturing gradient gel electrophoresis (DGGE) and terminal restriction fragment length polymorphism (T-RFLP) after PCR [16], [17], as well as community profiling based on fatty acid methyl esters (FAME) and phospholipid fatty acid (PLFA) [18]. Next-Generation Sequencing (NGS) is a new DNA sequencing method, which relies on the detection of pyrophosphate release upon nucleotide incorporation, rather than chain termination with dideoxynucleotides. Compared with DGGE and T-RFLP, NGS may provide more detailed information about the community because each DNA molecule is sequenced as an individual read and because the identification of individual species group is more accurate [19]. Of NGS, both Pyrosequencing and Illumina Miseq are used for characterize soil microbial community structure including rhizosphere microbiome [16], [20], however in recent studies, Illumina Miseq platform has been more frequently used since the around 10-fold increase in read depth, similar sequencing quality together with much lower cost [21].

Growth of fruit plants is affected by soil enzyme activity [22], [23], and soil enzyme activity is highly related to soil microbes [24], which are involved in nutrient cycling and plant nutrient availability, and are in turn influenced by plant species. Sun et al. [25] used pyrosequencing to characterize the bacterial community structure of apple rhizosphere soil with different manure ratios, finding that certain levels of manure treatment resulted in significantly higher soil enzyme activity and a more diverse bacterial community composition. Past surveys of microbial communities of apple replant sites in specialized growing areas in Europe [13], Australia [26] and the USA [2] have confirmed a complex of biotic pathogens as causal agents of this etiology. It is also suggested that genotype-specific interactions with soil microbial consortia are linked with apple rootstock tolerance or susceptibility to ARD [27], while some CG series dwarfing rootstock showed high tolerance to replant disease compared to M26 [28]. However, apple orchards in China have significant differences compared with other areas in the world, including soil with low organic matter content and the specific rootstock, such as Pingyitiancha, which is commonly used in the Bohai Bay region, one of the two leading apple-producing areas in China. Little is known about the status of soil microbial communities in ARD orchards in China. For this reason, a specific survey of bacterial community development in the rhizosphere of apple trees at a replant disease site and a new planting site was conducted with the following objectives: (i) to compare tree growth rate and soil enzyme activity between a replant disease site and a new planting site, (ii) to reveal bacterial community development under ARD and non-ARD conditions and (iii) to define the relative importance of biotic components in replant disease etiology.

## Methods

### Ethics statement

The experiment was carried out in our scientific research field for pomology studies which is owned by our institute, therefore, no specific permissions were required for these locations/activities, and the field studies did not involve endangered or protected species.

### Soil sampling

The test soil was collected from an orchard operated by the Institute of Pomology and Forestry, Beijing Academy of Agricultural Sciences. The soil type of local area was sandy loam soil, while the pH was 6.0–6.5. The orchard site was originally planted with apple trees approximately 1985 with trees grafted on *Malus Robusta* rootstock. In September 2012, the old trees were removed, and in April 4, 2013, the soil from a depth of 0–20 cm was collected and mixed as replant soil sample (*ReSoil*). Another soil sample from alongside the orchard intensively cultivated with vegetables was collected as a new plant soil sample (*NewSoil*). Soil was put into 64 liter cubic Plexiglas boxes with rainproof shelter, and the nursery was planted right after the soil sampling. In late September, soil from a depth of 0–20 cm in three different locations at 20-cm distances from the center of trunk was collected from both sites and the youngest part of roots and the adjacent soil were resampled as *RePlant* and *NewPlant*.

### Rootstock variety and tree growth

The two-year-old apple saplings were planted on April 4, 2013. Trees were of the scion variety ‘Fuji’ and were first grafted onto SH6 inter stock and then onto *Malus hupehensis* Var. Pingyiensis Jiang rootstock. The rootstock Pingyiensis Jiang has been widely used in Chinese orchards since the 1970 s. Trees were planted into either the replant soil or the new plant soil, with 10 replicates/treatment, and trunk heights were handed to 1.0–1.2 m. On September 20, 2013, new shoots and new roots of these trees were collected separately, dried at 105°C for 30 min and then dried at 70°C until a constant weight was reached in a forced-air oven.

### Soil enzyme activity characterization

Soil samples were collected from depths of 0–20 cm in three different locations at 20-cm distances from the center of nurseries using a 5-cm diameter soil auger and transferred on ice to the laboratory both before nursery planting, and at the beginning of autumn 2013, just after the growth of autumn-shoot ceased. The soil samples were sieved through a 2-mm screen and homogenized prior to the analysis. One portion of the composite soil was stored in DNA-free polythene bags and kept on dry ice for the molecular analysis, while another portion was used for enzyme activity measurements.

Soil urease, invertase and cellulase activities were estimated according to a previous report [25]. Soil urease activity was detected using improved sodium phenate and sodium hypochlorite colorimetry. Invertase and cellulase activities were estimated colorimetrically by determining the reduction of 3,5-dinitrosalicylic acid from reducing sugars after the soil was incubated with a buffered sucrose and sodium carboxymethylcellulose solution and toluene at 37°C for 24 h and 72 h, respectively.

### Soil DNA extraction

Three replicate samples were randomly picked for one treatment and used for DNA extraction. Soil DNA was extracted from the 1 g of soil after sieving using a Fast DNA SPIN Kit for soil (MP Biomedicals, Santa Ana, CA), according to the manufacturer’s instructions. The extracted soil DNA was dissolved in 100 µl TE buffer, quantified by NanoDrop and stored at −70°C before use.

### Bacterial 16S rRNA gene amplification and Illumina Sequencing

To determine the diversity and composition of the bacterial communities in each of these samples, we used the protocol described in Caporaso et al. [29]. PCR amplifications were conducted in with the 515f/806r primer set that amplifies the V4 region of the 16S rDNA gene. The primer set was selected as it exhibits few biases should yield accurate phylogenetic and taxonomic information. The reverse primer contains a 6-bp error-correcting barcode unique to each sample. DNA was amplified following the protocol described previously [30]. Amplicon pyrosequencing was performed on the Illumina MiSeq platforms at Novogene Bioinformatics Technology Co., Ltd, Beijing, China. Complete data sets are submitted to the NCBI Short Read Archive under accession no. SRX337490.

Pairs of reads from the original DNA fragments were merged by using FLASH [29] -a very fast and accurate software tool which was designed to merge pairs of reads when the original DNA fragments were shorter than twice the length of reads. Sequencing reads was assigned to each sample according to the unique barcode of each sample. Sequences were analyzed with the QIIME [31] software package (Quantitative Insights Into Microbial Ecology) and UPARSE pipeline [32], in addition to custom Perl scripts to analyze alpha (within sample) and beta (between sample) diversity.

First, the reads were filtered by QIIME quality filters. Default settings for Illumina processing in QIIME was used (r = 3 p = 0.75 total read length; q = 3; n = 0).

(p) min_per_read_length: minimum number of consecutive high-qualitybase calls to retain read(as percentage of totalread length).

(r) max_bad_run_length: maximum number of consecutive low-quality base calls allowed before truncating a read.

(n) sequence_max_n: maximum number of ambiguous (N) characters allowed in a sequence.

(q) phred_quality_score: last quality score considered low quality.

Then we use UPARSE pipeline to picking operational taxonomic units (OTUs) through making OTU table. Sequences were assigned to OTUs at 97% similarity. We picked a representative sequences for each OTU and used the RDP classifier [33] to assign taxonomic data to each representative sequence. In order to compute Alpha Divesity, we rarified the OTU table and calculated three metrics: Chao1 metric estimates the richness, the Observed OTUs metric was simply the count of unique OTUs found in the sample, and shannon index. Rarefaction curves were generated based on these three metrics.

### Statistical and bioinformatics analysis

Heatmap figures were generated using custom R scripts. Canoco 4.5 was used to run principal component analysis (PCA). Analysis of variance and Spearman's rank correlations were performed using SPSS Statistics 18 (IBM, Armonk, New York, USA). The community richness index, community diversity index, data preprocessing, operational taxonomic unit-based analysis and hypothesis tests were performed using mothur (http://www.mothur.org/). The histogram was created using Microsoft Excel 2010 (Microsoft, Redmond, Washington, USA). Significance was accepted at p<0.05, unless otherwise noted.

## Results

### Seedling biomass accumulation

In the spring of 2013, dwarfing apple nurseries of ‘Fuji’/SH6/Pingyitiancha trees were planted a replant site (*RePlant*) and a new planting site (*NewPlant*). Before planting, soil from the replant site (*ReSoil*) and from the new planting site (*NewSoil*) was sampled. After 1 year of growth, significant differences (P<0.05) were found between *RePlant* and *NewPlant* sites in dry mass accumulation (**Table 1**). Seedling growth was significantly inhibited in replant soil comparing with non-replant soil; the inhibition levels on root dry weight and shoot dry weight were 49.4% and 42.3%, respectively.

**Table 1:** The invertase, urease and cellulase activity of soil of different treatments and plant growth mass of *RePlant* and *NewPlant*.

	Invertase	Urease	Cellulase	New shoots (DW, g)	New roots (DW, g)
*RePlant*	8.93±0.93^c^	0.73±0.06^a^	0.164±0.032^b^	150.5±22.9	22.3±6.2
*NewPlant*	6.59±0.14^b^	1.02±0.22^b^	0.053±0.015^a^	261.4±34.6	44.1±9.7
*ReSoil*	8.87±0.22^c^	0.55±0.18^a^	0.182±0.064^b^	NA	NA
*NewSoil*	5.16±0.65^a^	0.60±0.13^a^	0.073±0.040^a^	NA	NA

“DW” means dry weight. Averages of replicates ± standard error; means followed by different letters are significantly different at *P*<0.05.

NA: not applicable.

### Soil enzyme activity

Soil urease activity was highest in *NewPlant* soil, whereas there was no significant difference in urease activity in soil from *RePlant*, *ReSoil* and *NewSoil* samples, which were 28.4%∼46.1% lower than *NewPlant* soil (**Table 1**). Cellulase activities in *NewPlant* and *NewSoil* were lower than in *RePlant* and *ReSoil*, but planting apple nurseries did not significantly change soil cellulase activity. Regardless of whether a new apple nursery had yet been planted, the soil from the old orchard had about three-fold higher cellulase activity than soil from a site that had never been planted. Invertase activity was lower in *NewSoil* and *NewPlant* samples than in *RePlant* and *ReSoil* samples.

### Richness

**Figure 1:**
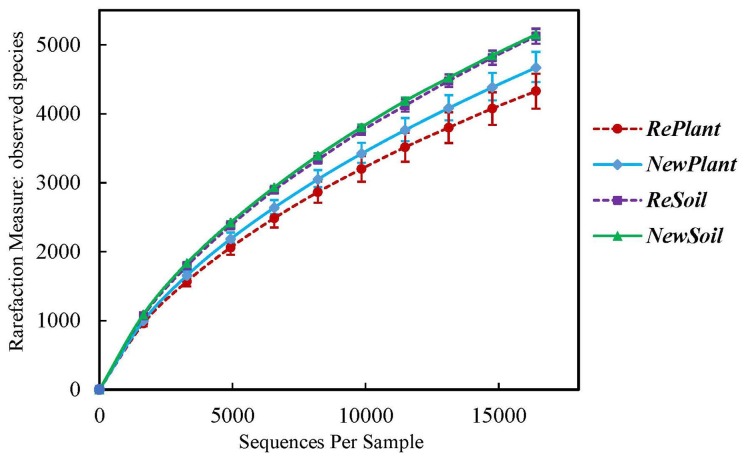
Rarefaction on species-abundance data. Average value of 3 replicates and error bar were showed.

More than 16,000 valid reads were obtained for each replicate through a sequence optimization process, and the bacterial community richness index was calculated as shown in **Table 2**. After quality filtering, median sequence length of each read was 252 bp. In *ReSoil* and *NewSoil* samples, more than 600 additional OTUs were observed compared with *RePlant* and *NewPlant* soil (**Fig. 1**). Higher Shannon and Chao 1 indices before planting indicated that planting of apple nurseries reduced diversity within the bacterial community. However, Pielou’s evenness values were indicating approximately equally distributed OTU abundances among community members (**Table 2**).

**Table 2:** Comparison of the estimated operational taxonomic unit (OTU) richness, diversity indexes and Pielous evenness of the 16S rRNA gene libraries for clustering at 97% identity as obtained from the pyrosequencing analysis.

Treatments	Observed OTUs	Shanon	Chao1	Pielous evenness (%)
*RePlant*	4328.1^a^	10.53^a^	9010.8^a^	87.17^a^
*NewPlant*	4509.9^a^	10.67^a^	9942.7^ab^	88.09^a^
*ReSoil*	5125.8^b^	11.02^b^	11371.8^c^	89.45^a^
*NewSoil*	5176.9^b^	11.12^b^	10614.9^bc^	88.53^a^

Averages of replicates ± standard error; means followed by different letters are significantly different at *P*<0.05.

### Taxonomic coverage

**Figure 2:**
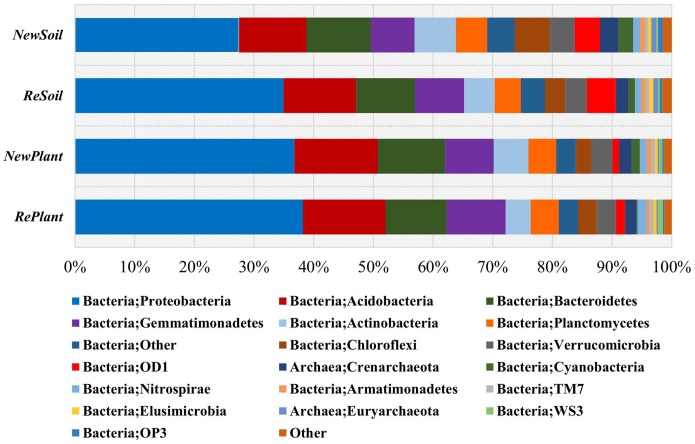
Comparison of the bacterial communities at the phylum level. Relative read abundance of different bacterial phyla within the different communities. Sequences that could not be classified into any known group were labeled “Other”.

All of the sequences were classified into 26 phyla or groups by the mothur program. The overall bacterial composition of the different samples was similar, while the distribution of each phylum or group varied (**Fig. 2**). In all samples, Proteobacteria, Acidobacteria, Bacteroidetes, Gemmatimonadetes and Actinobacteria were the five most dominant phyla, accounting for >60% of the reads. Significantly more unclassified species were detected in *ReSoil* and *NewSoil* samples, which was in accordance with their higher diversity indices. Compared with other samples, *NewSoil* had a significantly higher percentage of GN02 (2.7–5.3-fold), OP3 (1.8–3.1-fold), Chloroflexi (1.8–2.4-fold), and Verrucomicrobia (1.1–1.3-fold), and a lower percentage of Proteobacteria (*NewSoil*: 27.2%, *ReSoil*: 34.9%, *RePlant*: 38.2% and *NewPlant*: 36.8%). The percentages of CyanoBac and Verrucomicrobia were lowest in *RePlant*. More Firmicutes were detected in *NewPlant* than *RePlant* or *ReSoil* samples, and the lowest level was found in *NewSoil*. The OD1 group was approximately two- to three-fold higher in *RePlant* and *NewPlant* compared with *ReSoil* and *NewSoil*.

On a genus level, all 260 detected genera were shared by the four samples, except for *Chryseobacterium,* which was not detected in *ReSoil*, and *Marinobacter*, which was not detected in *RePlant*. Several cold-tolerant species belong to *Chryseobacterium* [34], while *Marinobacter* has been reported to be halophilic and is found in seawater [35].

**Figure 3:**
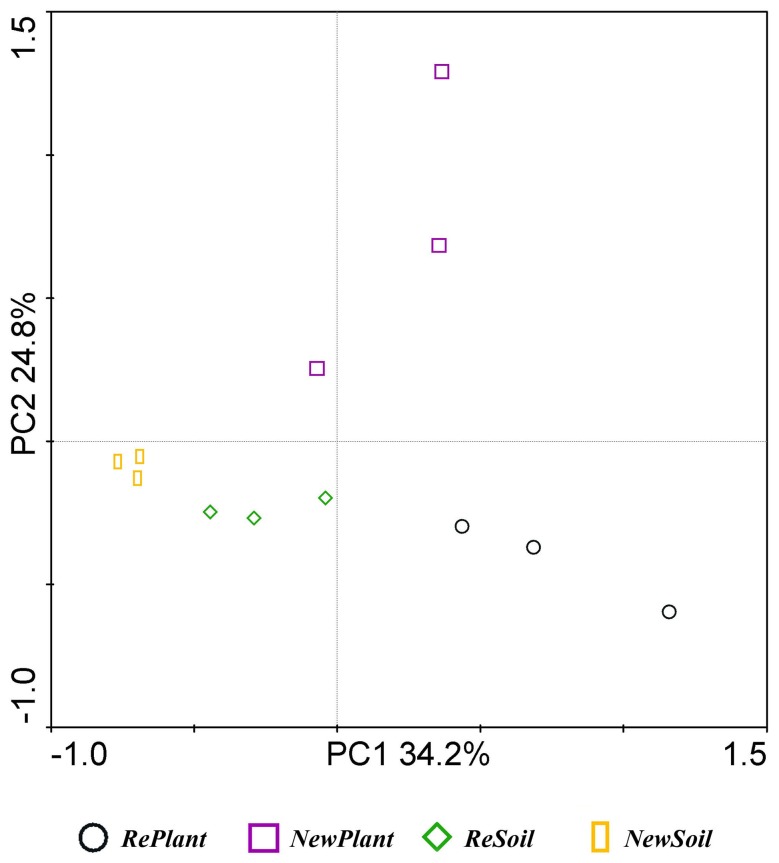
Principal component analysis (PCA) on the relative abundance of bacterial genera using Canoco 4.5. Principal components (PCs) 1 and 2 explained 34.2% and 24.8% of the variance, respectively.

To further compare the microbiota among the different samples, we performed PCA on the relative abundance of bacterial genera using Canoco 4.5 (**Fig. 3**). Data are presented as a 2D plot to better illustrate the relationship. *ReSoil* and *NewSoil* were relatively similar, but planting of an apple nursery had a significant impact on the soil microbial community. *RePlant* had a significantly higher PC1 value, and *NewPlant* had a higher PC2 value. 32 genera showing significant differences among the samples are listed in **Table 3**.

**Table 3:** The genera showing significant differences among the samples.

Taxon	*RePlant* (%)	*NewPlant* (%)	*ReSoil* (%)	*NewSoil* (%)
*Pseudomonas*	0.207±0.048^b^	0.120±0.041^a^	0.063±0.031^a^	0.057±0.009^a^
*Lysobacter*	0.941±0.260^b^	0.471±0.065^a^	0.276±0.061^a^	0.277±0.037^a^
*Phenylobacterium*	0.337±0.007^c^	0.258±0.045^b^	0.317±0.022^c^	0.135±0.013^a^
*Ramlibacter*	0.276±0.128^a^	0.728±0.109^b^	0.345±0.061^a^	0.336±0.046^a^
*Chthonomonas*	0.051±0.013^a^	0.171±0.048^b^	0.12±0.055^ab^	0.202±0.038^b^
*Flavisolibacter*	0.217±0.060^a^	0.847±0.350^b^	0.364±0.095^a^	0.354±0.048^a^
*Opitutus*	0.378±0.022^a^	0.400±0.057^a^	0.644±0.120^b^	0.524±0.060^ab^
*Bdellovibrio*	0.096±0.014^a^	0.161±0.058^ab^	0.256±0.025^b^	0.239±0.031^b^
*Novosphingobium*	0.185±0.136^ab^	0.266±0.049^b^	0.063±0.018^a^	0.058±0.009^a^
*Planctomyces*	0.167±0.025^a^	0.152±0.032^a^	0.195±0.032^ab^	0.239±0.002^b^
*Kaistobacter*	2.463±0.555^b^	2.302±0.461^b^	2.17±0.311^b^	0.978±0.083^a^
*Cellvibrio*	0.124±0.173^a^	0.100±0.064^a^	0.144±0.051^a^	0.378±0.06^b^
*Kribbella*	0.012±0.012^a^	0.031±0.011^a^	0.026±0.004^a^	0.081±0.025^b^
*Pseudonocardia*	0.035±0.014^a^	0.045±0.004^ab^	0.045±0.028^ab^	0.095±0.034^b^
*Rubrobacter*	0.118±0.066^a^	0.226±0.082^ab^	0.144±0.023^a^	0.311±0.092^b^
*Luteimonas*	0.388±0.071^b^	0.268±0.158^ab^	0.325±0.052^ab^	0.120±0.029^a^
*Steroidobacter*	0.902±0.136^ab^	0.701±0.085^a^	0.996±0.104^b^	0.761±0.065^ab^
*Candidatus Nitrososphaera*	1.282±0.400^a^	2.054±0.372^ab^	2.04±0.357^ab^	3.044±0.683^b^
Cenarchaeaceae;g__	0.476±0.180^b^	0.043±0.043^a^	0.061±0.064^a^	0.017±0.010^a^
Hyphomonadaceae;g__	0.721±0.078^b^	0.360±0.069^a^	0.646±0.055^b^	0.382±0.117^a^
Rhodospirillaceae;g__	0.721±0.104^c^	0.348±0.012^a^	0.577±0.099^bc^	0.455±0.129^ab^
Haliangiaceae;g__	1.520±0.475^b^	0.793±0.028^a^	0.811±0.143^a^	0.879±0.108^a^
Syntrophobacteraceae;g__	0.914±0.046^b^	0.614±0.212^a^	0.563±0.095^a^	0.587±0.051^a^
Rhodothermaceae;g__	0.033±0.018^a^	0.102±0.037^b^	0.033±0.013^a^	0.031±0.016^a^
Saprospiraceae;g__	0.366±0.114^a^	0.671±0.142^b^	0.378±0.054^a^	0.807±0.093^b^
Erythrobacteraceae;g__	0.553±0.150^ab^	0.469±0.080^a^	0.815±0.229^b^	0.309±0.042^a^
Polyangiaceae;g__	0.033±0.015^a^	0.061±0.044^a^	0.073±0.016^a^	0.145±0.012^b^
NB1-j;g__	0.341±0.056^b^	0.157±0.113^a^	0.083±0.044^a^	0.108±0.013^a^
Chromatiales;g__	0.106±0.025^b^	0.020±0.020^a^	0.047±0.029^a^	0.027±0.003^a^
Sphingomonadales;g__	0.159±0.048^ab^	0.240±0.015^c^	0.220±0.043^a^	0.099±0.009^a^
Sphingomonadaceae;g__	0.679±0.076^a^	1.026±0.234^b^	0.689±0.064^a^	0.423±0.051^a^
Micrococcaceae;g__	0.183±0.062^a^	0.563±0.167^b^	0.228±0.013^a^	0.673±0.105^b^

“g__” represents genus not grouped into any known genera within these families/groups. Averages of replicates ± standard error; means followed by different letters are significantly different at *P*<0.05.

*Pseudomonas, Lysobacter* and *Phenylobacterium* were significantly higher in *RePlant* compared with *NewPlant*, while *Ramlibacter, Chthonomonas* and *Flavisolibacter* were higher in *NewPlant*. *Phenylobacterium* and *Kaistobacter* were higher in *ReSoil* compared with *NewSoil*, while *Cellvibrio, Kribbella* and *Rubrobacter* were higher in *NewSoil*. On both the replant site and the new plant site, apple nurseries showed significant impacts on the bacterial community structure. On the new plant site, *Phenylobacterium, Ramlibacter, Flavisolibacter, Novosphingobium* and *Kaistobacter* had increased abundance, and *Planctomyces, Cellvibrio* and *Kribbella* decreased in abundance. On the replant site, only *Pseudomonas* and *Lysobacter* increased, while *Opitutus* and *Bdellovibrio* decreased. Compared with the *NewPlant* sample, planting in the replant site also resulted in a greater abundance of certain genera of Cenarchaeaceae, Hyphomonadaceae, Rhodospirillaceae, Haliangiaceae and Syntrophobacteraceae families, however these genera weren’t grouped into known genera within these families.

## Discussion

Replanted soil significantly inhibited root and shoot development and exhibited different soil enzyme activity and a different bacterial community pattern. The levels of inhibition on root dry weight and shoot dry weight were 49.4% and 42.3%. Rosette disease and decreasing photosynthetic efficiency were also observed in the replant site, as were fewer main branches (data not shown). This observation is in accordance with the negative impact of replanting on apple growth that is widely reported [9], [13], [36].

Soil enzymes are involved with biological cycling and the development of fertility, so they are crucial indicators of the soil biochemistry. Urease catalyzes the hydrolysis of urea to produce ammonia and carbamate, and it is thus recognized as an important indicator of soil health. In this study, rhizosphere soil of *NewPlant* showed significantly increased urease activity, but rhizosphere soil of RePlant did not, indicating that in new planting sites, the root exudates might support a new and different functional microbial community which was responsible for this apparent increase in mineralization and result in a better supply of available nutrients. This result was supported by data on plant dry mass (**Table 1**), where *NewPlant* exhibited more than 40% greater plant growth compared with *RePlant*. Xun et al. [22] reported that soil urease activity increased during apple orchard maturation, and in our previous study on manure refinement of apple orchards, urease was the key indicator of soil health and highly correlative to tree growth, no matter whether the soil type was sandy [24] or loam [25]. Therefore, soil urease is an ideal indicator of apple orchard maturation and the lack of a significant increase in urease activity at the replant site may explain the decreased growth and late fruiting. However, soil invertase, which is an important factor affecting hydrolysis of sucrose into glucose and fructose, and cellulase, which is involved in breaking down cellulase, were higher in replant soil rather than new soil, and planting of apple nurseries had no further impact on these two enzyme activities. This could be related to the residual small root tissues of previous trees. Invertase also increased with an overdose of manure refinement in apple orchards, but was not closely associated with tree growth [37].

The soil microbial community composition of the replant site and the new plant site were distinct, and planting of an apple nursery significantly increased the difference. The diversity within the bacterial community was reduced after planting an apple nursery in both replanted and newly planted soil, which was in accordance with the fewer unclassified species observed after nurseries had been growing for one year. It has been reported that many plant species reduce the microbial diversity of rhizosphere soil compared with surrounding sites, including maize [38] and switchgrass [39]. Such a reduced bacterial richness in the plant rhizosphere is known as the ‘rhizosphere effect’. This is typically characterized by a selective enrichment of root specialized guilds and reduction of rhizosphere bacterial richness in comparison to unplanted soil. However, although the diversity at both sites decreased after planting, there was no significant difference in bacterial diversity between the replant and new plant sites, either before or after the nursery had been planted, which means the rhizosphere effect of apple trees is the critical factor determining bacterial community diversity.

Although Proteobacteria, Acidobacteria, Bacteroidetes, Gemmatimonadetes and Actinobacteria predominated in all of the samples, *NewSoil* had a unique phyla composition compared with the other samples. Higher percentages of GN02, OP3, Chloroflexi and Verrucomicrobia and lower percentages of Proteobacteria were observed. This could also be explained by the rhizosphere effect’ because there must be some root tissue left in the *ReSoil* site, even without a new nursery being planted. It is worth noticing that the OD1 group was approximately two- to three-fold higher in the *RePlant* and *NewPlant* samples as compared with *ReSoil* and *NewSoil*, and the *RePlant* soil had a higher percentage of WS3 compared with the other treatments. Verrucomicrobia were less common in the *RePlant* sample compared with *NewPlant*. Cultivation-independent approaches detect representatives of the Verrucomicrobia phylum in a wide range of environments, including soils, water and human feces [40], suggesting the phylum is widespread, but it is still poorly characterized. A few species that are extremely acidophilic [41] or ectosymbionts of protists [42] belong to this phylum. In our previous study [25], on loam soil of apple orchards with manure refinement, an optimal manure ratio resulted in an increase of Verrucomicrobia compared with soil with no manure applied, with the abundance increasing from 1.10% to 1.51%. However, on sandy soil [24], Verrucomicrobia decreased monotonically from 2.62% to 1.71%, 1.36% and 0.97% following application of 5%, 10% and 15% (which was optimal for tree growth) manure, respectively, and increased back to 1.28% when 20% manure applied. More research is required to determine whether this phylum is related to the rhizosphere effect of apple trees. Because most species of GN02, OP3, OD1 and WS3 groups are known only from metagenomics study and remain uncultivated, little information is available for functional discussion.

Principal component analysis on the genus level showed that apple nurseries had significant impacts on the soil microbial community, and the changes from *ReSoil* to *RePlant* and from *NewSoil* to *NewPlant* differed significantly. The bacterial community difference between *RePlant* and *NewPlant* was much greater than between *ReSoil* and *NewSoil*.

*Phenylobacterium* and *Kaistobacter* were higher in *ReSoil* compared with *NewSoil*, while *Cellvibrio, Kribbella* and *Rubrobacter* were higher in *NewSoil*. The genus *Phenylobacterium* comprises a single species called *P. immobile*, which has previously been described as growing optimally only on artificial compounds such as chloridazon [43]. More *P. immobile* in replant soil could be related to herbicide applied in the orchard. Unfortunately, little information is available in the literature concerning the genus *Kaistobacter*. *Cellvibrio* is a genus of gamma proteobacteria, which can oxidize cellulose to oxycellulose, but *NewSoil* showed lower cellulase than *ReSoil*, probably because in an aerobic environment, soil cellulase mainly derives from fungi, rather than bacteria. Some species of *Kribbella* have been isolated from the rhizosphere of *Typhonium giganteum* [44] and from tissues such as apricot leaves [45] and roots of *Lupinus angustifolius* [46]. *Kribbella antibiotic* [47] of the genus was reported to have a strong inhibitory activity toward *Botrytis* sp., *Rhizoctonia solani* and *Pyricularia oryzae*. *Rubrobacter* is a genus of Actinobacteria, which are radiotolerant [48], and a novel DNA repair enzyme was isolated from *Rubrobacter radiotolerans* [49], but little information is available regarding its presence in orchard soil.

On the replant site, *Lysobacter* and *Pseudomonas* increased in abundance, while *Opitutus* and *Bdellovibrio* decreased. *Pseudomonas* and *Lysobacter* were significantly higher in *RePlant* compared with *NewPlant*, while *Ramlibacter, Chthonomonas* and *Flavisolibacter* were more abundant in the *NewPlant* sample. *Pseudomonas* has been proposed to play a role in replant disease etiology of peach [50] and apple [51] trees through the production of hydrogen cyanide (HCN). However, *Pseudomonas* also includes several soil bacterial species with plant growth promoting activity, including *P. fluorescens* [10], [52], and *Pseudomonas* and *Bacillus* are two of the most common biocontrol agent sources [53]. *Lysobacter* [54] has been a rich source for novel antibiotics, and some species have potential as biological control agents for plant diseases. Considering the previous study of manure refinement of apple orchard, in which an optimal manure ratio for nursery growth resulted in a decrease of *Pseudomonas* and *Lysobacter* [24], [37], we speculate that in the *RePlant* sample, the higher percentage of *Pseudomonas* was related to replant disease and the increase of *Lysobacter* and was probably induced by the high percentages of Pathogenic fungi. However, further culture-based experiments would be needed to confirm this. There was only one species of *Chthonomonas*, *G. calidirosea*, which is an aerobic, pigmented, thermophilic micro-organism [55], while two species of *Flavisolibacter* were isolated from ginseng cultivating soil [56]. However, little was known about their function in soil or their relationship with planting until now.

Compared with *NewPlant*, planting in the replant site also resulted in more of certain genera of Cenarchaeaceae, Hyphomonadaceae, Rhodospirillaceae, Haliangiaceae and Syntrophobacteraceae families; however, these genera weren’t grouped into any known genera within these families. This is in accordance with the fact that pyrosequencing can detect many uncultured microbes.

## Conclusions

Our study has documented that replanting has a large negative impact on growth of apple nurseries in Beijing, China. Planting apple nurseries raised soil urease activity at the new planting site but not the replant site, while no significant impact on invertase and cellulase was observed. Apple nurseries had a significant impact on the soil bacterial community. *Lysobacter* and *Pseudomonas* were increased at the replant site, and the bacterial communities of the new and replant sites responded differently, resulting in more distinct community patterns.
